# The clinical efficacy of epalrestat combined with α-lipoic acid in diabetic peripheral neuropathy: Protocol for a systematic review and meta-analysis

**DOI:** 10.1097/MD.0000000000009828

**Published:** 2018-02-09

**Authors:** Xiaotong Wang, Haixiong Lin, Shuai Xu, Yuanlin Jin, Ren Zhang

**Affiliations:** a Shenzhen Bao’an Traditional Chinese Medicine Hospital Group, Guangzhou University of Chinese Medicine, Shenzhen; b The First School of Clinical Medicine; c School of Chinese Materia Medica; d The College of Fundamental Medical Science, Guangzhou University of Chinese Medicine, Guangzhou, People's Republic of China.

**Keywords:** α-lipoic acid, diabetic peripheral neuropathy, epalrestat, meta-analysis, protocol, systematic review

## Abstract

**Background:**

Diabetic peripheral neuropathy (DPN) is a common long-term complication of diabetes mellitus, affecting patients in the world. Epalrestat combined with α-lipoic acid (ALA) is the most frequent combine therapy used in the DPN researches. We aim to assess the effectiveness and safety of epalrestat combined with ALA in patients with DPN, compare with epalrestat alone.

**Methods:**

We will search Cochrane Library, PubMed, Wanfang Data, China National Knowledge Infrastructure, VIP Chinese Science and Technology Journals Database, and Chinese Biomedical Database from inception until October 31th, 2017. Inclusion the randomized controlled trials and clinical control trials of combine therapy which evaluate clinical efficacy and side effect in people with DPN. Data extraction and risk of bias assessments will be independently conducted by 2 reviewers. The primary outcome measures will be total effective rate, motor nerve conduction velocity (MNCV), sensory nerve conduction velocity (SNCV), Toronto clinical scoring system (TCSS), and total symptom score (TSS). All statistical analyses will be performed using RevMan V.5.3 software.

**Results:**

This review will evaluate the total effective rate, nerve conduction velocity, TCSS, TSS, and safety of ALA combined with epalrestat for patients with DPN, compare with epalrestat alone.

**Conclusion:**

Our study will provide evidence to assess whether epalrestat combined with ALA is an optional treatment for patients with DPN.

## Abstract 2

Supplemental Digital Content is available in the text

## 1 Introduction

Diabetic peripheral neuropathy (DPN) is a common long-term complication of diabetes mellitus, affecting approximately 50% of diabetic patients worldwide.^[1]^ Recently, Toronto Diabetic Neuropathy Expert Group provided a definition for DPN^[2]^: “Typical DPN, also known as chronic distal symmetrical polyneuropathy is a symmetrical, length-dependent sensorimotor polyneuropathy attributable to metabolic and microvessel alterations as a result of chronic hyperglycemia exposure and cardiovascular risk covariates.” Therefore, the typical symptoms of DPN are pain, burning, tingling, cramps, paresthesia, and numbness.^[3]^ DPN is connected to increased mortality correlative to its severity.^[4]^ Patients with DPN also have complications such as foot ulcers, osteomyelitis, osteoarthropathy (Charcot foot), medial arterial calcification, and as well as neuropathic edema, which diminished the quality of life.^[5,6]^ An European prospective study of 4400 participants found the prevalence of DPN raise from 7.5% at diagnosis to 45% after 25 years.^[7]^ These data highlight the critical need to seek out an effective treatment for this condition. According to the oxidative stress and related pathways of DPN, some drugs are produced and wisely used in clinical treatment, such as taurine, acetyl-L-carnitine, aldose reductase inhibitors (epalrestat, ranirestat), α-lipoic acid (ALA), protein kinase C inhibitor (ruboxistaurin), inhibitor of poly ADPribose polymerase (nicotinamide), angiotensin-converting enzyme inhibitor (trandolapril), advanced glycation end product inhibitors (aspirin, benfotiamine), etc.^[8]^ However, in order to achieve better therapeutic effect, more and more people are inclining to combine medication.^[9]^ And several systematic reviews suggested that combine medication, such as methylcobalamin plus prostaglandin E1, or lipoic acid plus methylcobalamin, was promising for managing DPN than monotherapy.^[10,11]^ However, no previous relevant systematic review concerning epalrestat combined with ALA, compare with epalrestat alone, in DPN has been planned or performed yet. Additionally, there is inadequate evidence in favor of the widespread use of epalrestat combined with ALA. Therefore, the aim of our study is to assess current clinical studies connected to the efficacy and safety of epalrestat combined with ALA versus epalrestat as a treatment for patients with DPN.

## 2 Materials and methods

### 2.1 Study type

We plan to include the randomized controlled trials and clinical control trials that assessed the effectiveness or side effect of ALA combined with epalrestat in people with DPN. We will exclude cohort studies, cross-sectional studies, reviews, comments, and animal experiments. We will not impose any race, sex, age, region, duration of patients, or severity of disease restrictions on the studies included.

### 2.2 Participants

Patients with DPN will be included in this systematic review. DPN should be confirmed according to the standard diagnostic criteria including the statement of American Diabetes Association, or the guidelines for prevention and treatment of type 2 diabetes (2013 version) of the Chinese Diabetes Society. The diagnostic criteria for diabetes mellitus are consistent with the criteria of the 1999 World Health Organization. We will exclude studies of patients with other types of peripheral neuropathy like Guillain–Barre syndrome, cerebral infarction, cervical spondylosis, severe venous vascular disease, lumbar lesion, and so on.

### 2.3 Interventions

Intervention group involving the combination of epalrestat and ALA is eligible. Control group will receive epalrestat alone. We will exclude treatment strategies that are not repeated.

### 2.4 Outcome measures

The primary outcomes include: total effective rate, motor nerve conduction velocity (MNCV), sensory nerve conduction velocity (SNCV), Toronto clinical scoring system (TCSS), and total symptom score (TSS). Total effective rate should according to the following criteria: subjective symptom was alleviated, tendon reflex was improved, and nerve conduction velocity was increased by more than 3 m/s after treatment.

The secondary outcomes are adverse reactions, gastrointestinal reaction.

### 2.5 Data sources

We will search the following databases from inception to October 31th 2017: Cochrane Library, PubMed, Wanfang Data, China National Knowledge Infrastructure, VIP Chinese Science and Technology Journals Database, and Chinese Biomedical Database. The following text terms will be used to find eligible trials: epalrestat, thioctic acid, lipoic acid, diabetic peripheral neuropathy, DPN, randomized controlled trial, trial, and blind. The details of search strategies that will be applied to the China National Knowledge Infrastructure and PubMed are presented in the Supplementary File 1. Other electronic databases will be search using the similar strategy. We will also search reference lists of relevant studies for potential eligible clinical trials. The languages will be limited to Chinese and English.

### 2.6 Study selection and data extraction

**Figure 1:**
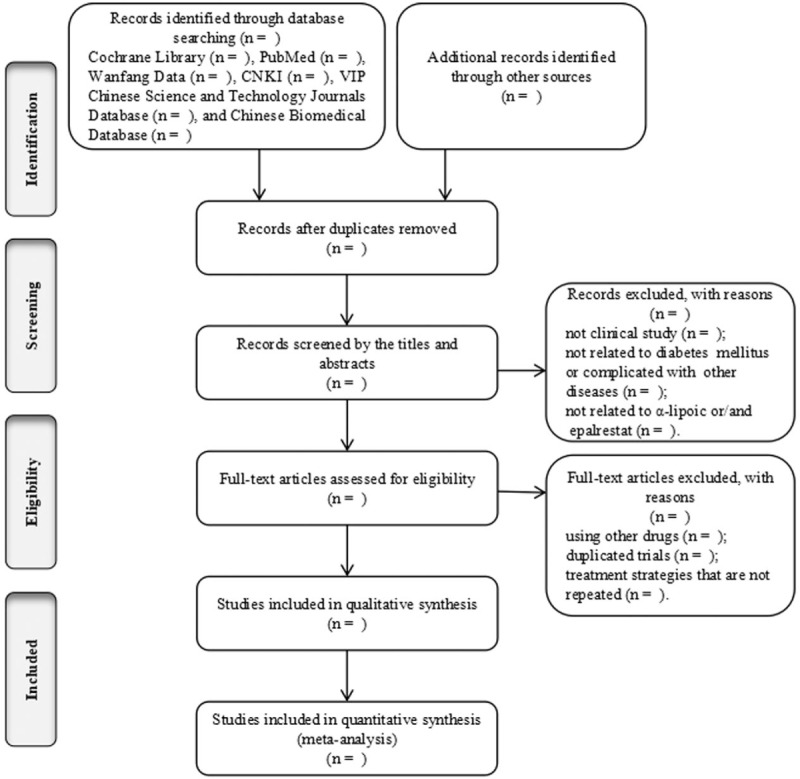
Flow chart of the search process.

We will import all retrieved data into NoteExpress V.3.2.0 and remove duplicate data from different databases. Two reviewers (XT Wang, HX Lin) will use the criteria described above to independently scan the abstracts and full texts to select potential references. Selection process will be presented in a Preferred Reporting Items for Systematic review and Meta-Analysis flow chart (http://www.prisma-statement.org/) (Fig. 1). Then, XT Wang, HX Lin will extract study information, such as first author names, publication year, sample sizes, gender, intervention methods, treatment duration, outcome, and follow-up periods. S Xu will check the final dataset. Discrepancies will be settled by consensus among the authors (XT Wang, HX Lin, YL Jin, and R Zhang).

### 2.7 Addressing missing data

In the case of missing data, we will contact the authors of the studies through email or telephone to obtain more additional information. If sufficient data cannot be accept in this way, we will analyze the available data from the trials and pay attention to the potential impact of inadequate information on the results.

### 2.8 Risk of bias in included studies

The quality of each eligible study included in this review will be checked using the bias assessment tool in Cochrane Handbook. This risk assessment contains 7 parts, such as random sequence generation, allocation concealment, blinding of participants and personnels, blinding of outcome assessments, incomplete outcome data, selective reporting, and other source of bias. In the end, the risk of bias will be divided into 3 levels: high, unclear, and low.

### 2.9 Data synthesis and analysis

Meta-analysis will be conducted in RevMan V.5.3 software. Dichotomous variable data will be presented as relative ratio or odds ratio with 95% confidence interval. Continuous variable data will be presented as standard mean difference or weighted mean difference with 95% confidence interval for the included studies. The chi-square test and *I*^2^ values will be used to assess the statistical heterogeneity.^[12]^*I*^2^ value ≤25% is regarded as no heterogeneity, *I*^2^ value ≥75% is considered significant heterogeneity, and 50% ≤*I*^2^ <75% indicated mild significant heterogeneity.^[13]^ According to the guidelines of Cochrane review, heterogeneity of *I*^2^ ≥50% warrants the use of the random effects model. Otherwise, a fixed-effects model is proper.

### 2.10 Additional analyses

If enough subgroup studies exist, subgroup analysis will be carried out to distinguish heterogeneity between subgroups. Subgroup analysis criteria are as follows:

(1) Duration of medicine treatment.
(2) Duration or severity of DPN.

### 2.11 Assessment of reporting biases

Reporting biases will be checked by funnel plots. If asymmetry is showed by a visual inspection, Egger's test or Begg's test will be used to further analyze the potential publication bias. A *P*-value >.05 in Egger's test or Begg's test reveal no significant publication bias.

### 2.12 Quality of evidence

We will also assess the quality of evidence for primary outcomes with the Grading of Recommendations Assessment, Development and Evaluation approach.^[14]^ The limitations of study design, inconsistency, indirect evidence, inaccuracy, and publication bias will be considered. This classifies the evidence into 4 levels: high, moderate, low, or very low.

### 2.13 Ethics and dissemination

We aim to explore current evidence connected with the effectiveness and safety of epalrestat combined with ALA in DPN, compared with epalrestat monotherapy. The main outcome includes total effective rate, MNCV, SNCV, TCSS, TSS, and adverse reactions. At last, the results of this review will be disseminated by a peer-reviewed journal. Ethical assessment does not require because only existing sources of literature will be included and evaluated.

## 3 Discussion

DPN is the most common neuropathic syndrome occurs on patients with diabetes. Diabetic damage due to hyperglycemia and metabolic imbalance, primarily oxidative stress, may appear in the neurons (axons or myelin sheaths) of DPN patients.^[8]^ Some related pathways like poly-ADP-ribose polymerase, advanced glycation end products, polyol, protein kinase C, and hexosamine originated from initial oxidative stress.^[15]^ Additionally, oxidative stress results in endothelial cell damage and vascular dysfunction, consequently caused peripheral nerve ischemia and hypoxia.^[16]^ To date, most of the treatment strategies were based on the related pathways of DPN. However, no drugs can completely cure it.^[8]^ Research reported that the efficacy of monotherapy is still not satisfied.^[9]^ Furthermore, the total annual economic burden linked to DPN and its complications is estimated at 4.6 to 13.7 billion dollars in the USA.^[17]^ These factors lead many patients to seek more effective treatment to manage DPN, like combination therapy which is recognized more effective than monotherapy. Most recent studies have reported on the use of combination therapy, such as epalrestat plus methylcobalamine, epalrestat combined with ALA, but the effects are controversial when compare with monotherapy.^[18,19]^ Some reviews have already been published suggested that combination therapies, such as methylcobalamin plus prostaglandin E1, or lipoic acid plus methylcobalamin, were more effective than monotherapy for patients with DPN.^[10,11]^ However, no previous relevant systematic review regarding the effectiveness and safety of epalrestat combined with ALA, compare with epalrestat, in DPN has been planned or published yet, even though epalrestat combined with ALA is frequently used in DPN.^[19,20]^

Study has proved that epalrestat could reduce the expression of antioxidant enzymes and aldose reductase, alleviate oxidative stress, and suppress the polyol pathway.^[21]^ ALA could directly eliminate free radicals, inhibits peroxidation, enhances blood flow, increases nerve Na^+^–K^+^ ATPase activity, and protects endothelial function.^[22]^ The aim of this systematic review is to evaluate the clinical effect of epalrestat combined with ALA on total effective rate, MNCV, SNCV, TCSS, and TSS, and safety of DPN in patients. In particular, we will distinguish various treatment strategies that are used in DPN according to the different dosage and time of treatment. In order to ensure the accuracy and reliability of the results, treatment strategies that are not repeated will be deleted. We plan to use adequate evidence to assess the clinical efficacy and safety of epalrestat combined with ALA in patients with DPN, compare with epalrestat. Our findings should be of benefit to practitioners in the fields of combining therapy.

## Supplementary Material

Supplemental Digital Content
