# Supplementary material for: The clinical efficacy of epalrestat combined with α-lipoic acid in diabetic peripheral neuropathy: Protocol for a systematic review and meta-analysis
Source: Medicine (Baltimore). 2018 Feb 9;97(6):e9828. doi: 10.1097/MD.0000000000009828 (PMC5944676; doi:10.1097/MD.0000000000009828)
Supplement: Supplemental Digital Content [file medi-97-e9828-s001.doc]

**Epalrestat Combined with Alpha lipoic acid in Diabetic Peripheral Neuropathy**

**Xiaotong Wang, MD & MS^1*^, Haixiong Lin, MD & MS^2*^, Shuai Xu, BS^3^, Yuanlin Jin, MD & MS^1^, Ren Zhang, PhD^4^**

**1** Shenzhen Bao’an Traditional Chinese Medicine Hospital Group, Guangzhou University of Chinese Medicine, Shenzhen 518133, People's Republic of China

**2** The First School of Clinical Medicine, Guangzhou University of Chinese Medicine, Guangzhou 510405, People's Republic of China

**3** School of Chinese Materia Medica, Guangzhou University of Chinese Medicine, Guangzhou 510006, People's Republic of China

**4** The College of Basic Medical Science, Guangzhou University of Chinese Medicine, Guangzhou 510006, People's Republic of China

^*^ These authors contributed equally to this work.

Correspondence to:

Yuanlin Jin, MD & MS, The Bao’an District Traditional Chinese Medicine Hospital Group, the Affiliated Hospital of Guangzhou University of Chinese Medicine, NO.25 Yu’an second Road, Bao’an district, Shenzhen 518133, People's Republic of China. ([1807759191@qq.com](mailto:1807759191@qq.com)).

Ren Zhang, PhD, Department of Microbiology, College of Fundamental Medical Science,Guangzhou University of Chinese Medicine, NO.232 Waihuan Dong Lu, Guangzhou Higher Education Mega Center, Guangzhou, 510006, People's Republic of China. (E-mail: zhangrenn@foxmail.com, Fax:+8602039358007, Tel:+8613751855281).

Represents the search strategy for PubMed, CNKI.

Search strategy used in PubMed.

| Number | Search terms |
| --- | --- |
| #1 | thioctic [All Fields] |
| #2 | acid [All Fields] |
| #3 | #1 and #2 |
| #4 | lipoic [All Fields] |
| #5 | acid [All Fields] |
| #6 | #4 and #5 |
| #7 | Alpha lipoic acid [MeSH] |
| #8 | Alpha lipoic acid [TIAB] |
| #9 | Alpha lipoic acid [All Fields] |
| #10 | lipoic acid [MeSH] |
| #11 | lipoic acid [TIAB] |
| #12 | lipoic acid [All Fields] |
| #13 | thioctic acid [MeSH] |
| #14 | thioctic acid [TIAB] |
| #15 | thioctic acid [All Fields] |
| #16 | #3 or #6 or #7 or #8 or #9 or #10 or #11 or #12 or #13 or #14 or #15 |
| #17 | diabetic [All Fields] |
| #18 | peripheral neuropathy [All Fields] |
| #19 | #17 and #18 |
| #20 | neuropathy [All Fields] |
| #21 | #17 and #20 |
| #22 | diabetic neuropathy [All Fields] |
| #23 | diabetic peripheral neuropathy [All Fields] |
| #24 | diabetic neuropathy [MeSH] |
| #25 | diabetic peripheral neuropathy [MeSH] |
| #26 | diabetic neuropathy [TIAB] |
| #27 | diabetic peripheral neuropathy [TIAB] |
| #28 | #19 or #21 or #22 or #23 or #24 or #25 or #26 or #27 |
| #29 | epalrestat [MeSH] |
| #30 | epalrestat [Supplementary Concept] |
| #31 | epalrestat [All Fields] |
| #32 | epalrestat [TIAB] |
| #33 | #29 or #30 or #31 or #32 |
| #34 | randomized [MeSH] |
| #35 | randomized controlled trial [MeSH] |
| #36 | randomized controlled trial [All Fields] |
| #37 | randomisation [MeSH] |
| #38 | #34 or #35 or #36 or #37 |
| #39 | blind [MeSH] |
| #40 | #16 and #28 and #33 and #38 and #39 |

Search strategy used in CNKI.

| Number | Search terms |
| --- | --- |
| #1 | 糖尿病 [主题] |
| #2 | 神经病变 [主题] |
| #3 | #1 and #2 |
| #4 | 糖尿病 [全文] |
| #5 | 神经病变 [全文] |
| #6 | #4 and #5 |
| #7 | 糖尿病 [关键词] |
| #8 | 神经病变 [关键词] |
| #9 | #7 and #8 |
| #10 | 糖尿病周围神经病变[主题] |
| #11 | 糖尿病周围神经病变[关键词] |
| #12 | 糖尿病周围神经病变[全文] |
| #13 | #3 or #6 or #9 or #10 or #11 or #12 |
| #14 | 硫辛酸[主题] |
| #15 | 硫辛酸[关键词] |
| #16 | 硫辛酸[全文] |
| #17 | 硫辛酸胶囊[全文] |
| #18 | 硫辛酸注射液[全文] |
| #19 | #14 or #15 or #16 or #17 or #18 |
| #20 | 依帕司他[主题] |
| #21 | 依帕司他[关键词] |
| #22 | 依帕司他[全文] |
| #23 | #20 or #21 or #22 |
| #24 | #13 and #19 and #23 |
